# Exploration of the minimal clinically important difference value of the 3‐min simulated pedal motion in patients with chronic obstructive pulmonary disease: A self‐controlled prospective clinical trial

**DOI:** 10.1111/crj.13687

**Published:** 2023-08-16

**Authors:** Xiaoting Zhang, Ni Liu, Feng Yang, Guansheng Su, Jieying Hu, Rongchang Chen, Zeguang Zheng

**Affiliations:** ^1^ Zhengzhou Central Hospital Affiliated to Zhengzhou University Zhengzhou Henan China; ^2^ State Key Laboraory of Respiratory Disease, National Clinical Research Center for Respiratory Disease, Guangzhou Institute of Respiratory Health The First Affiliated Hospital of Guangzhou Medical University Guangzhou Guangdong China; ^3^ Shenzhen Institute of Respiratory Diseases Shenzhen City Guangdong province China

**Keywords:** 3MSPM, 6MWD, chronic obstructive pulmonary disease, exercise therapy, pulmonary rehabilitation

## Abstract

**Background:**

To help elderly patients with severe or very severe chronic obstructive pulmonary disease (COPD) with pulmonary rehabilitation, we have developed Zheng's supine rehabilitation exercise (ZSRE). Currently, none of the terminal or critically ill patients with severe exercise limitation can complete the 6‐min walking distance (6MWD) and cardiopulmonary exercise testing (CPET).

**Methods:**

In this study, we discuss the definition of the standardized 3‐min simulated pedal motion (3MSPM) test and its operational specifications. Also, we evaluate the minimal clinically important difference (MCID) value of the 3MSPM.

**Results:**

The results showed that the mMRC score of COPD patients with acute exacerbation of dyspnea was progressively reduced from the second day of respiratory rehabilitation, and the difference between the first and seventh days was statistically significant (*p* < 0.000, *χ* ^2^ = 176.664). 6MWD increased progressively, and the difference between 6MWD on day 1–7 was statistically significant (*p* = 0.024, *F* = 2.443). The difference between 3MSPM on day 1–7 was also statistically significant (*p* < 0.000, *F* = 4.481). Further analysis showed that 6MWD was negatively correlated with mMRC (*p* < 0.000, *OR* = −0.524). 3MSPM was positively correlated with 6MWD (*p* < 0.000, *OR* = 0.640) but negatively correlated with mMRC (*p* < 0.000, *OR* = −0.413). There is a linear regression relationship between 6MWD and 3MSPM, that is, 6MWD = 14.151 + 0.301 * 3MSPM, adjusted *R* ^2^ = 0.401.

**Conclusion:**

Based on the regression equation, 3MSPM can predict 6MWD, and it can be used as a simple exercise endurance method to evaluate patients with safety hazards in underground activities or who cannot complete the 6MWD test. The minimum clinically important difference value is increased by 23.

## Graphical abstract

Our results indicate based on the regression equation, 3MSPM can predict 6MWD, and it can be used as a simple exercise endurance method to evaluate patients with safety hazards in underground activities or who cannot complete the 6MWD test. The minimum clinically important difference value is increased by 23.

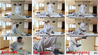

## 1 INTRODUCTION

Chronic obstructive pulmonary disease (COPD) is one of the risk factors for high disability rate and mortality rates worldwide.^1^ Pulmonary rehabilitation cannot improve pulmonary ventilation but can improve skeletal muscle weight and its muscle strength and endurance, improve exercise tolerance and quality of life, and reduce acute exacerbations and their frequency of hospitalization in COPD patients.^1^, ^2^ In order to help elderly patients with severe or extremely severe COPD to perform pulmonary rehabilitation, we developed Zheng's supine rehabilitation exercise (ZSRE), which includes three movements: stretching and sitting, bridge exercise, and simulated pedal motion, which has the following 4S advantages: (1) simple: easy to learn and suitable for elderly patients; (2) satisfy: trains upper limb, lower limb, back, and abdominal muscles; (3) safe: can be performed in bed and patients can stop resting at any time according to their condition; (4) save: not limited by time and space and can be performed during hospitalization and at home. Currently, neither the 6‐min walking distance (6MWD) nor the cardiopulmonary exercise test (CPET) can be completed for terminal or critically ill patients with severe exercise limitation, making it difficult to assess the rehabilitation effect. Our previous study found that ZSRE could be performed in elderly patients with acute exacerbation of chronic obstructive pulmonary disease (AECOPD) in GOLD stage IV without serious adverse events.^3^ In view of the rehabilitation effectiveness and safety of ZSRE and the increase in simulated pedal motion per unit time as exercise endurance improves, we constructively suggest that simulated pedal motion can be used not only as a form of rehabilitation exercise for patients but also as an indicator of exercise endurance. In this study, we discuss the definition of the standardized 3‐min simulated pedal motion (3MSPM) and its operational specifications. In addition, we evaluated the minimal clinically important difference (MCID) values of 3MSPM.

## 2 METHODS

### 2.1 Subjects and methods

#### 2.1.1 Subjects

Patients with AECOPD who were admitted to the Department of Pulmonary and Critical Care Medicine (PCCM) at the First Hospital of Guangzhou Medical University from August 1, 2018, to February 28, 2019, were selected, and patients were recruited according to the following inclusion and exclusion criteria.

Inclusion criteria:

- Inpatients with AECOPD who met clinical discharge targets.
- Age: 40–80 years, no gender restriction.
- The patient had a history of smoking and pulmonary ventilation test results within 6 months: FEV1/FVC ratio <0.70 and FEV1 <80% of normal expected values after bronchodilator administration.
- Able to complete the standardized 3MSPM test, Standard Intensive Exercise Training (SIET) and the 6MWD test.
- Signed the written informed consent form.

Exclusion criteria:

- Poorly controlled blood pressure, with systolic blood pressure (BPsys) >200 mmHg and or diastolic blood pressure (BPdia) >100 mmHg.
- Resting heart rate (HR_rest_) greater than 130 bpm.
- Resting SpO_2_ <88% without oxygen inhalation; or peripheral oxygen saturation ≤90% with oxygen therapy or noninvasive ventilation.
- Deep vein thrombosis without indwelling filters, especially in the lower extremities or pelvic veins.
- Acute phase of connective tissue diseases.
- The patient has been treated with pulmonary rehabilitation.
- An event of unstable angina or myocardial infarction, left atrial thrombosis or acute left ventricular insufficiency within 1 month.
- Patients who are unable to complete the submaximal exercise test.
- Significant illness or condition other than COPD that is not considered appropriate for SIET by a clinician, respiratory therapist, or rehabilitation therapist.
- Patients who refused to sign the informed consent form or who could not tolerate SIET training.

#### 2.1.2 Pulmonary rehabilitation

All patients received 7 days of pulmonary rehabilitation prior to discharge. The rehabilitation prescription was personalized according to the patient's dyspnea, fatigue symptoms, or SIET. Prescriptions included total body exercise training, respiratory muscle training, and airway secretion clearance.

#### 2.1.3 Observation markers

Patients' MMRC scores and 6MWD were assessed daily at 8:00 a.m. The interval between the 6MWD test and the 3MSPM test was at least 1 h. Daily assessments were followed by SIET.

#### 2.1.4 Experiment procedure

1. Baseline assessment

After screening for eligible patients, their resting heart rate (HR), respiratory rate (RR), and dyspnea score (BORG) were measured as baseline values: heart rate (HR_base_), respiratory rate (RR_base_), and dyspnea score (BORG_base_).

2 Blood gas analysis and pulmonary ventilation function were tested: FVC, FEV1, and FEV1/FVC.
3 SIET prescriptions.

- Total body movement exercise

**FIGURE 1:**
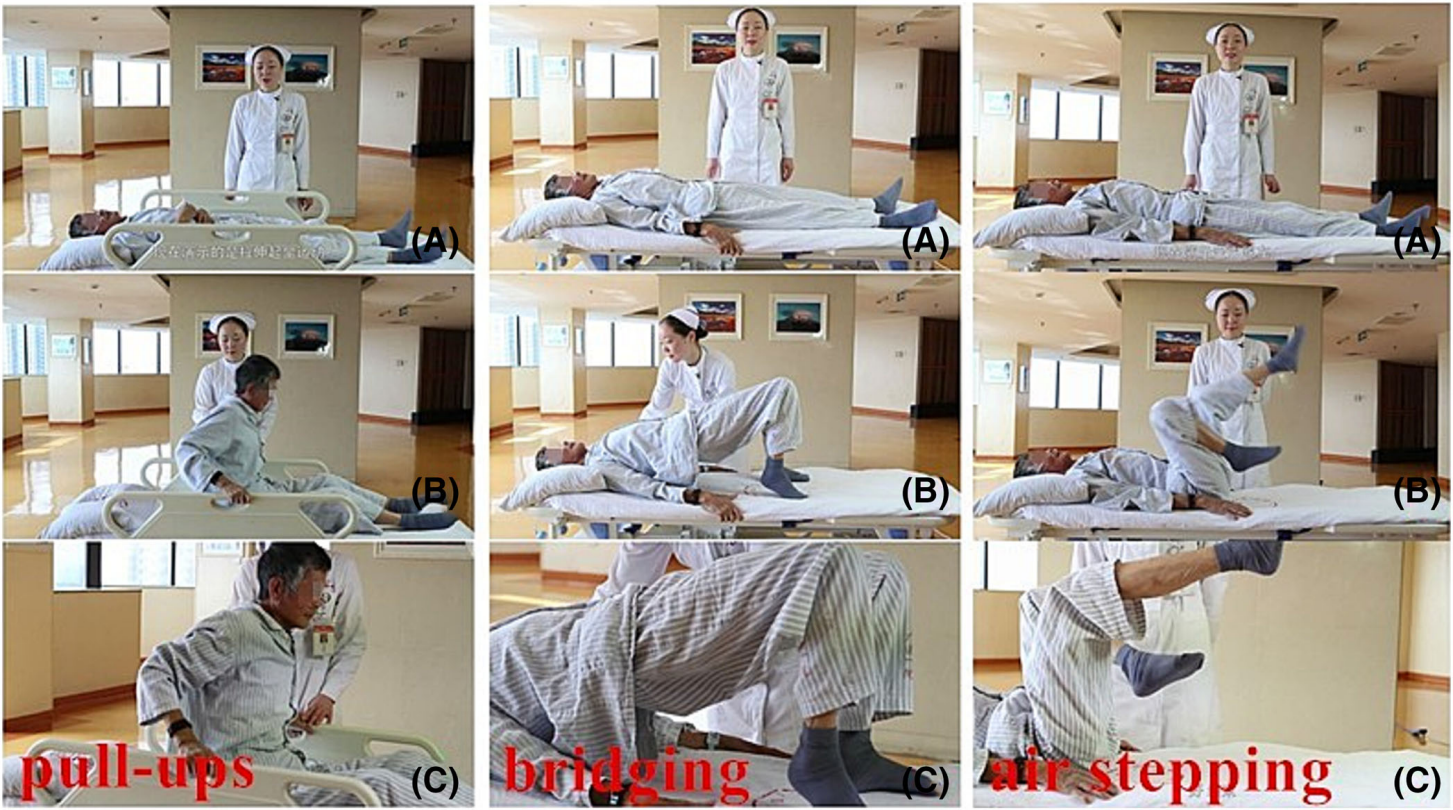
Zheng's supine rehabilitation exercise.

ZSRE was used as a whole‐body exercise training, and ZSRE consisted of three movements: air bicycle, bridge exercise, and stretching, each exercised for 5 min and repeated at least 20 times until the patient's BORG score was no less than 5 (Figure 1).

b Inspiratory muscle training

Belly exercise with 2.5 kg of heavy load around the umbilicus.

c Expiratory muscle and airway secretion clearance

The respiratory trainer with oscillatory positive expiratory pressure (OPEP) function was used. The expiratory threshold resistance of this device is 25 cmH_2_O and synchronous shock can be generated simultaneously at the expiratory phase. Expiratory muscles can be exercised, and airway secretions can be cleared through forceful inhalation and exhalation through this trainer to overcome resistance. Each exercise consists of 10 sets; each set consists of blowing the respiratory trainer five times, with a 2‐ to 3‐s interval between each set. Active coughing to clear airway secretions was also carried out.

d The above rehabilitation components were performed twice daily and could be performed under nasal cannula oxygen inhalation at 3 L/min. Patients were guided by comfort to complete the above exercise tasks, except for whole‐body exercise which required achieving a BORG score of no less than 5 points, and patients could stop and rest briefly at any time.
e SIET termination marker

① Heart rate ≥130 bpm or ≤50 bpm.

② RR ≥35 bpm or ≤12 bpm.

③ Arterial oxygen saturation ≤88%.

④ BPsys ≥200 mmHg or ≤90 mmHg, BPdia ≥110 mmHg or ≤50 mmHg, or a change in BP greater than 50% of baseline.

⑤ Ischemic changes requiring management of cardiac rhythm disturbances or low cardiac ST pressure.

⑥ The patient feels uncomfortable and asks for termination.

4 Vital signs monitoring

During SIET, respiratory rate, heart rate, peripheral oxygen saturation, and ECG were continuously monitored; blood pressure was measured for each completed element, and Borg scores were tested at the beginning, 1.5 min, and end of 3MSPM.

5 Testing of observation markers
  - Standardized test methods for 3MSPM

The patient lies supine on a horizontal bed with both lower limbs off the bed, left hip and knee at an angle of 90°, lower leg parallel to the bed, and right lower limb straightened. The left lower limb extends straight toward the distal end of the bed and the lower limb leaves the bed and maintains an angle of 30° with the bed, while the right lower limb is flexed so that the angle between the hip and knee is 90°. The above exercises are repeated alternately for the left and right lower limbs until fatigue, which could be repeated after rest. A standard simulated pedal movement is defined as achieving an angle of 90° between the hip and knee to full extension. During the 3‐min test, patients were encouraged to complete as many movements as possible. The total number of completed exercises was the result of the 3MSPM test. To ensure that the 3MSPM was maximized, the first element of the SIET was the simulated pedal motion that was carried out each morning, and the number of completions in the previous 3 min was used as the 3MSPM test result.

b Testing of 6MWD

Testing was carried out according to the methods specified in the ATS Clinical Lung Function Laboratory Proficiency Standards Committee guidelines.^4^

c mMRC assessment

Dyspnea severity was rated using the Modified Medical Research Council (mMRC) Dyspnea Scale with a scale of 0–4. Grade 0: I only get breathless with strenuous exercise; Grade 1: I get short of breath when hurrying on level ground or walking up a slight hill; Grade 2: On level ground, I walk slower than people of the same age because of breathlessness, or have to stop for breath when walking at my own pace; Grade 3: I stop for breath after walking about 100 m or after a few minutes on level ground; Level 4: I am too breathless to leave the house or I am breathless when dressing.

d BORG Assessment

The BORG dyspnea scale uses a scale of 0–10, with 0: not at all, “nothing” meaning that there was no effort, no muscle fatigue, no shortness of breath or difficulty breathing; 0.5: just noticeable (extremely slight, just noticeable); 1: very slight (“very slight” means very little effort, at your own pace, and you are willing to walk closer); 2: slight (“mild”); 3: moderate (means some but not great difficulty. Feels like it is okay and not difficult to continue); 4: somewhat severe; 5: severe (“intense‐severe”, very difficult, strenuous, but not very difficult to continue). This level is about half of the “maximum”; 6: between 5 and 7; 7: very severe (“very strong” you can continue, but you have to force yourself and you are extremely tired); 8: between 7 and 9; 9: very, very severe (almost maximal); 10: maximal (“very intense‐maximum” is an extremely intense level, and for the vast majority of people it is the most intense they have ever experienced in their lives.).

e Standardized Intensive Training (SIET) movements

1. Stretching and sitting action: The patient grasps the edge of the bed with his/her hands and uses the strength of both upper limbs to pull the upper body to an upright sitting position, hold for 5 s, then slowly lie down.
2. Bridge movement: The patient lies on his back, knees bent, feet placed flat on the bed, hands on the umbilicus, and then actively raise the hips to about 10–15 cm from the bed.
3. Simulated pedal motion: same as 3MSPM.

f Periumbilical load exercise and diaphragm movement: A 2.5‐kg sandbag was placed around the umbilicus; the subject was instructed to inhale forcefully to inflate the abdomen and maintained for 2–3 s before relaxing and exhalation.
g Respiratory trainer movements: Inhale forcefully and then exhale, gradually increase the force of the exhale until an oscillation occurs, then maintain until the end of exhalation.
h Method for determining the MCID value of 3MSPM

1. Based on the ROC curve, the MCID values of 3MSPM were determined by 6MWD before intensive exercise workout and mMRC improvement separately; where 37, 54, and 71 m were used for 6MWD and 1 point for mMRC improvement.
2. A regression relationship between 3MSPM and 6MWD was explored based on the regression equation of 3MSPM and 6MWD.

### 2.2 Statistical analysis

Inter‐group differences in quantitative data were tested by one‐way analysis of variance (ANOVA), and rank data was tested using Friedman test. Differences between 3MSPM/6MWD/MMRC and day 1 data were tested by paired *t* test or Wilcoxon test. Correlations between 3MSPM, 6MWD and mMRC were testing using Spearman's test. MCID values for the 3MSPM test were corrected using correction methods such as ROC curves or regression equations. A difference with *p* < 0.05 differences was considered to be statistically significant.

## 3 RESULTS

### 3.1 Collection of patient information

Between August 1, 2018, and February 28, 2019, 229 patients with AECOPD were hospitalized, 158 met the recruitment criteria and participated in the 7‐day rehabilitation, the rehabilitation study was terminated in 11 patients, and finally 147 including 67 patients in GOLD A group, 71 patients in GOLD B group, and nine patients in GOLD E group completed the 7‐day rehabilitation study (see Table 1).

**TABLE 1:** General condition of patients.

	*n* = 147	χ±s
Gender (male/female)	108:39	
Age (years)		65.85 ± 15.68
Disease of respiratory system	24	
Pulmonary heart disease	25	
Other circulatory diseases	4	
Hypertension	2	
Diabetes	2	
Chronic nephritis	13	
Central nervous system disease	17	
Baseline heart rate (bpm)		89.12 ± 15.68
Resting respiratory rate (cycles/min)		22.56 ± 6.72
Resting BP_sys_ (mmHg)		121.36 ± 23.87
Resting BP_dia_ (mmHg)		72.43 ± 23.87
PCO_2_ (mmHg)		43.40 ± 15.76
PO_2_/FiO_2_ (mmHg)		344.86 ± 120.19
FEV_1_ (%)		61.13 ± 26.70
FEV1/FVC (%)		56.55 ± 26.56
FVC (%)		62.62 ± 48.84
MMEF_25_ (%)		42.20 ± 27.99

Abbreviations: BP_sys_, systolic blood pressure; BP_dia_, diastolic blood pressure; FEV_1_, forced expiratory volume in 1 s; FVC, forced vital capacity; HR, heart rate; MMEF_25_, 25% of maximum midexpiratory flow25%.

### 3.2 Adverse events and serious adverse events

Twenty‐three patients were suspended from the 6MWD walking test due to Arterial oxygen saturation below 90%, and 18 of them completed the 6‐min walking test after oxygen inhalation. Therefore, the incidence of adverse events of hypoxemia was 14.56%. Six patients withdrew from the study because of severe arrhythmia including two patients with atrial tachycardia who were restored to sinus rhythm after medical treatment and four patients with sinus tachycardia whose heart rate was greater than 130 bpm who voluntarily withdrew from the study. The incidence of arrhythmia was 3.80%. The test was suspended in one patient because the systolic blood pressure exceeded 200 mmHg during the 3MSPM test due to missing anti‐hypertensive medication.

### 3.3 MMRC, 6MWD, 3MSPM, and BORG dynamic changes during intensive rehabilitation

1. mMRC dynamic changes

Beginning on day 2 of pulmonary rehabilitation, mMRC scores progressively decreased, with statistically significant differences in mMRC scores between days 1–7 (*p* < 0.000, *χ* ^2^ = 176.664) (see Table 2).

2 6MWD dynamic changes

A progressive increase in 6MWD began on day 2 of pulmonary rehabilitation, with a statistically significant difference between 6MWD on days 1–7 (*p* = 0.024, *F* = 2.443) (see Table 2).

3 3MSPM dynamic changes

The 3MSPM was 66.29 ± 40.89 on day 1 and decreased to 60.99 ± 26.45 on day 2 but continued to increase from day 3, with daily 3MSPM values from day 4 onwards being higher than those on day 1; the difference between days 1–7 3MSPM was statistically significant (*p* < 0.000, *F* = 4.481) (see Table 2).

4 BORG score dynamic changes

Borg score was the highest at 1.5 min of the 3MSPM test and lowest at the beginning of the test, with a statistically significant difference (*p* = 0.000); Borg scores before and after the 3MSPM test on days 1–7 were similar and not statistically different, but Borg scores at 1.5 min after the start of exercise on days 1–7 were highest on day 3 of training, with a statistically significant difference (see Table 3).

**TABLE 2:** Dynamic changes of 3MSPM/6MWD/MRC during intensive rehabilitation ( χ±s).

	Day1	Day2	Day3	Day4	Day5	Day6	Day7	p*	*F*/*χ* ^2^
3MSPM	66.29 ± 40.89	60.99 ± 26.45	67.79 ± 31.99^②②^	72.27 ± 30.09^①②②③③^	75.16 ± 26.29^①②②③^	79.33 ± 45.67^①①②②^	95.97 ± 39.73^①①②②③③④④⑤⑤⑥⑥^	<0.000^a^	4.481^a^
mMRC score	2.69 ± 1.11	2.69 ± 0.99	2.74 ± 0.83	2.64 ± 0.82	2.65 ± 1.01^①②③^	1.96 ± 0.67^①①③③④④⑤⑤^	1.85 ± 0.67^①①②②③③④④⑤⑤^	<0.000^b^	176.664^b^
6WMD	214.98 ± 155.08	228.76 ± 148.97	229.37 ± 157.18	240.73 ± 162.85	250.19 ± 148.04	302.77 ± 194.31	272.50 ± 169.43	0.024^c^	2.443^c^

Compared with Day1: ① *p* < 0.05, ①① *p* < 0.01.

Compared with Day2: ② *p* < 0.05, ②② *p* < 0.01.

Compared with Day3: ③ *p* < 0.05, ③③ *p* < 0.01.

Compared with Day4: ④ *p* < 0.05, ④④ *p* < 0.01.

Compared with Day5: ⑤ *p* < 0.05, ⑤⑤ *p* < 0.01.

Compared with Day6: ⑥ *p* < 0.05, ⑥⑥ *p* < 0.01.

Abbreviations: 3MSPM, 3‐min simulated pedal motion; 6MWD, 6‐min walking distance; mMRC, Modified Medical Research Council score.

^a^ One‐ANOVA test.

^b^ Paired sample *t* test.

^c^ Wilcoxcon's test.

**TABLE 3:** Dynamic change of BORG score during intensive rehabilitation ( χ±s).

The duration of intensive respiratory rehabilitation	Beginning	1.5 min	Ending	*p*	*χ* ^2^
Day1	4.78 ± 1.45	8.21 ± 0.94	6.31 ± 1.26	0.000	206.665
Day2	5.93 ± 0.84	7.78 ± 1.71	6.40 ± 1.16	0.000	158.653
Day3	5.44 ± 0.91	8.91 ± 1.44	6.40 ± 1.10	0.000	159.170
Day4	4.53 ± 0.88	7.97 ± 1.67	6.28 ± 1.25	0.000	138.567
Day5	4.59 ± 0.87	8.84 ± 1.19	6.47 ± 1.11	0.000	119.514
Day6	4.72 ± 0.85	8.78 ± 1.26	6.53 ± 1.05	0.000	103.507
Day7	4.94 ± 0.84	8.66 ± 1.29	6.41 ± 1.10	0.000	79.000
*p*	0.469	15.125	0.125		
*χ* ^2^	0.525	0.000	0.724		

*Note*: Borg scores were tested at the beginning, 1.5 min and end of 3MSPM, Friedman test.

### 3.4 MCID values for 3MSPM

There is a linear regression relationship between the 6MWD and the number of 3MSPM, and 6MWD could be calculated indirectly by the following formula: 6MWD = 14.151 + 0.301 * 3MSPM, adjusted for *R* ^2^ = 0.401. Based on ROC curves, 6MWD improvement of 37, 54, or 71 m was used as the minimal clinical difference value for exercise endurance improvement, and the difference between 6MWD values from day 6 of intensive rehabilitation exercise compared with day 1 was statistically significant; MCID values for 3MSPM was 23 for Δ6MWD of 37, 54, or 71 m on days 6–7, respectively, with a sensitivity of 0.680 and a specificity of 0.619; the corresponding AUC area value was 0.70 with 95% confidence interval (0.586–0.820). The MCID values for all 3MSPM determined by mMRC improvement of 1 score were 65.5 (see Tables 4 and 5).

**TABLE 4:** MCID values of 3MSPM determined by calibration method.

	AUC	AUC 95% CI	S.E	*p*	MCID	Sensitivity	Specificity
Standard 1(Δ6MWD = 54)	0.638	(0.521–0.550)	0.060	0.045	23	0.640	0.603
Standard 2 (Δ6MWD = 37)	0.703	(0.586–0.820)	0.060	0.003	23	0.680	0.619
Standard 3 (Δ6MWD = 71)	0.685	(0.578–0.792)	0.054	0.011	23	0.762	0.627
Standard 4 (mMRC = 1)	0.595	(0.535–0.655)	0.031	0.002	65.5	0.598	0.508

Abbreviations: 3MSPM, 3‐min simulated pedal motion; 6MWD, 6‐min walking distance; AUC, area under curve; MCID, the minimal clinically important difference.

**TABLE 5:** MCID value of 3MSPM determined by regression equation method.

	Adjusted *R* ^2^	*F*	*p*	Regression equation (Y = 6MWD, X = 3MSPM)
Linear equation	0.401	56.575	0.000	Y = 14.151 + 0.301X

*Note*: 6MWD was used as the calibration standard for the linear regression equation.

Abbreviations: 3MSPM, 3‐min simulated pedal motion; 6MWD, 6‐min walking distance; MCID, the minimal clinically important difference.

## 4 DISCUSSION

Patients hospitalized with AECOPD who met discharge indications after treatment but still had dyspnea were given intensive pulmonary rehabilitation therapy with sustained improvements in exercise tolerance and dyspnea markers such as 6MWD, 3MSPM, and mMRC scores. Compared with 6MWD, 3MSPM was more sensitive to the patients' exercise tolerance and its MCID value was 23. However, the above criteria are derived from data from recently discharged patients, and whether they are applicable to patients with stable COPD remains to be demonstrated.

Pulmonary rehabilitation is beneficial for improving exercise tolerance, improving shortness of breath,^5^, ^6^, ^7^ improving quality of life, and reducing acute exacerbations and hospitalizations.^1^ Pulmonary rehabilitation includes whole body muscle training, respiratory muscle training, airway clearance, nutritional therapy and elimination of causes or triggers of acute exacerbation, and mainly consists of rehabilitation training of exercise endurance. Rehabilitation effects are related to the intensity of exercise rehabilitation, the higher the intensity the better the rehabilitation effect. The mechanism of exercise training to improve exercise endurance may be related to the promotion of mitochondrial oxidative metabolism‐related gene enrichment,^1^ promotion of skeletal muscle NAMPT protein increase, and improvement in VO2max.^8^

Endurance exercise rehabilitation prescription for patients with chronic respiratory disease includes frequency, intensity, duration, and type of exercise SIET^1^, ^9^: Frequency is three to five times per week,^10^ intensity is Borg dyspnea or fatigue score of 4,^11^, ^12^ duration is 20–60 min per session, and type includes endurance training, resistance/strength training, flexibility training, interval training, upper body training, and inspiratory muscle training.

Based on the principles of SIET prescription, patients achieved a Borg dyspnea or fatigue score of 4 or more after exercise. The subjects of this study were elderly, severely impaired pulmonary function patients who met the discharge indication after hospitalization for acute exacerbation in the presence of dyspnea. To ensure safety during exercise rehabilitation, we developed a recumbent rehabilitation exercise, which consisted of three movements: simulated pedal motion, bridge exercise, and stretching. The recumbent exercise consists of three movements: simulated pedal motion, bridge exercise, and stretching and sit‐up. The whole‐body electromyogram collected during the recumbent rehabilitation exercise showed that the simulated pedal motion mainly involved lower limb muscle movements, the stretching and sit‐up involved biceps, triceps, pectoralis major and abdominal muscles, and the bridge exercise involved rectus abdominis muscle movements. We studied the safety of recumbent rehabilitation exercises (ZSRE) and their effectiveness in elderly patients with chronic obstructive pulmonary disease who were hospitalized for acute exacerbations over 60 years of age and with FEV1 (pre%) = 30.5 ± 12.1. The results^11^, ^12^ showed that 41 patients in the rehabilitation group performed ZSRE three times a day with 15–20 repetitions of each movement each time, and the results showed that all patients were able to complete ZSRE, and there were no unexpected events such as hypoxemia and abnormal heart rhythm. At the time of discharge, the CAT score, 6MWD distance and mMRC score of the rehabilitation group were better than those of the non‐rehabilitation group. After discharge, all the other 36 subjects in the rehabilitation group were able to adhere to home rehabilitation, except for five cases who were lost‐to‐follow‐up, the compliance rate was as high as 36/41, and there were no unexpected events. At the 8th week of discharge, the CAT score, 6MWD distance, and mMRC scores in the rehabilitation group improved. The results suggest that recumbent rehabilitation exercises are safe and beneficial for patients' motor endurance improvement and symptom improvement.^13^

In this study, patients were encouraged to use ZSRE exercise to achieve a Borg dyspnea score of 6 or more, the intensity of daily exercise rehabilitation was increased, and patients trained at a frequency of 2 sessions/day for 1 week to improve rehabilitation outcomes.

In this study, patients who met the discharge criteria but still had dyspnea were given 7 days of intensive pulmonary rehabilitation training before discharge, including: total body exercise, respiratory muscle rehabilitation training and airway clearance; total body exercise was performed by ZSRE including three movements of stretching and sitting, simulated pedal motion and bridge exercise, respiratory muscle rehabilitation included inspiratory shoulder shrugging and abdominal bulging movement, inspiratory resistance training, expiratory resistance training, and airway clearance using positive expiratory pressure oscillation (PEPO). Because the effect of whole‐body exercise rehabilitation is related to the intensity of rehabilitation, we aimed to achieve a BORG score of 6 or more after daily exercise. The results showed that the patients had a significant increase in BORG score after ZSRE, and all patients reached a score of 6 or more, suggesting that the intensity of exercise rehabilitation achieved the desired goal.

Although pulmonary rehabilitation improves dyspnea symptoms and improves exercise endurance, it does not improve pulmonary ventilation, so pulmonary ventilation function could not be used in the assessment of pulmonary rehabilitation. Instead, markers related to dyspnea, exercise endurance, respiratory symptoms, and quality of life were used, with exercise endurance being more objective in assessing the effect of pulmonary rehabilitation.

Commonly used assessments of dyspnea include BORG scale and mMRC dyspnea scale. The BORG scale assesses dyspnea during exercise to determine the exercise intensity of the test subjects. The mMRC dyspnea scale is used to retrospectively describe scenarios of dyspnea to assess the exercise endurance of the patient.

Exercise endurance is assessed by the exercise cardiopulmonary test, in which exercise is performed under a certain power load and the function of the heart, lungs, and skeletal muscles are assessed by measuring metabolic markers such as oxygen uptake and carbon dioxide output, ventilation markers, and electrocardiographic changes. The types of tests used to assess exercise endurance include: planking, pedaling, 6‐min walk test (6WMD), and stair climbing test. 6WMD is one of the most commonly used markers to assess exercise endurance, but is only indicated for patients who can tolerate walking and cannot be assessed in bedridden or critically ill patients.

Respiratory symptoms are assessed by the BCSS score, CAT score, of which the CAT score and the mMRC dyspnea scale are also tools for assessing the A‐D classification of COPD patients; quality of life was assessed by using the St. George's respiratory questionnaire (SGRQ).

The results of this study showed that in patients recovering from acute exacerbation of COPD who met discharge indication after treatment but still had dyspnea, the mMRC continued to improve every morning starting on day 5 of combined pulmonary rehabilitation when combined conventional medication. On days 6 and 7 of rehabilitation, the mMRC score decreased from at least 2 to less than 2, that is, group D to group C. However, the improvement in 6MWD before and after the pulmonary rehabilitation intervention was not statistically different, which is related to the short duration of rehabilitation in this study of only 7 days. Rehabilitation exercise to improve walking distance requires at least 5–8 weeks of rehabilitation.^13^, ^14^, ^15^ In addition, for older patients recovering from acute exacerbation of chronic obstructive pulmonary disease with severe impairment of pulmonary function, the use of 6MWD to assess patients' exercise endurance has disadvantages of insensitivity and false negatives, which are explained by the fact that 6MWD is not only influenced by the patient's cardiac, pulmonary, and skeletal muscle function but also by other factors such as the complexity of motor movements, coordination and their safety. There is no simple, safe, and sensitive exercise endurance assessment test for movement in patients with advanced age and severely impaired lung function with chronic obstructive pulmonary disease. The present study showed that the number of 3‐min simulated pedal motions (3MSPM) gradually increased from day 2 of pulmonary rehabilitation. From day 3, its increase was statistically significant and negatively correlated with the daily dynamic changes in mMRC values (*r* = 0.64 vs. *r* = 0.45), and the MCID value of 3MSPM was 65.5 when using the dyspnea index mMRC as a reference marker. After intensive rehabilitation, both 3MSPM and 6MWD improved progressively, with a positive correlation (*p* < 0.000, *OR* = 0.640), using an increase in 6MWD of 54, 37, or 71 m as the reference marker for the minimal clinically important difference value, respectively. 3MSPM MCID values for assessing improvement in exercise tolerance were both 23, where the area under the ROC curve was greatest when the reference marker was 34 m, with a sensitivity of 0.680 and specificity of 0.619. The results suggest that the improvement of 3MSPM can not only reflect the improvement in dyspnea, but also 3MSPM can sensitively respond to the exercise endurance of patients compared with 6MWD (Table 4), its movements are simple and safe to implement on bed. Hence, it can be used not only as a pulmonary rehabilitation method but also as a marker to assess exercise endurance in elderly and frail patients.^16^ At the same time, it can be inferred from the regression equation (14.151 + 0.301 * 3MSPM) that the patient could not complete 6MWD. However, due to the absence of a control group, the data from this study cannot confirm that the improvement in the patient's dyspnea mMRC is entirely an effect of pulmonary rehabilitation, and a rigorous multicenter controlled study is needed to facilitate the clinical dissemination of this intensive pulmonary rehabilitation method.

## 5 CONCLUSION

However, due to the absence of a control group, the data from this study cannot confirm that the improvement in the patient's dyspnea mMRC is entirely an effect of pulmonary rehabilitation, and a rigorous multicenter controlled study is needed to facilitate the clinical dissemination of this intensive pulmonary rehabilitation method. In addition, another study limitation is that our data were limited to patients who had recently been discharged from the hospital; we did not include patients with long‐term stable COPD or those who were hospitalized.

In conclusion, mMRC, 6MWD, and 3MSPM improved progressively after 7 days of intensive rehabilitation in chronic obstructive pulmonary patients recovering from an acute exacerbation in the presence of dyspnea, with 3MSPM showing a high correlation with both mMRC and 6MWD; 3MSPM can be used to deduce 6MWD and is a simple method of assessing exercise tolerance in patients with safety concerns about exercise on the ground or inability to complete the 6MWD test. Its clinical minimum is +23.

## AUTHOR CONTRIBUTIONS

**Xiaoting Zhang:** Data curation; writing—original draft preparation. **Ni Liu:** Conceptualization; methodology; writing—original draft. **Feng Yang:** Writing—review and editing. **Guansheng Su:** Visualization. **Jieying Hu:** Software; validation. **Rongchang Chen:** Supervision. **Zeguang Zheng:** Project administration; conceptualization; methodology.

## CONFLICT OF INTEREST STATEMENT

We declare that we have no conflict of interest.

## ETHICS STATEMENT

The study was approved by the First Affiliated Hospital of Guangzhou Medical University (Approval 2018/115). The clinical trial registration number is ChiCTR1900020529 (http://www.chictr.org/cn/).

## Data Availability

The data that support the findings of this study are available on request from the corresponding author. The data are not publicly available due to privacy or ethical restrictions.
